# Differential Risk Factor Profiles in the Prediction of General and Pain-Specific Functional Limitations 12 Months after Major Pediatric Surgery

**DOI:** 10.3390/children8050360

**Published:** 2021-04-30

**Authors:** Brittany N. Rosenbloom, P. Maxwell Slepian, M. Gabrielle Pagé, Lisa Isaac, Fiona Campbell, Jennifer Stinson, Joel Katz

**Affiliations:** 1 Department of Psychology, York University, Toronto, ON M3J 1P3, Canada; maxwell.slepian@uhn.ca; 2 Department of Anesthesia and Pain Management, Toronto General Hospital, University Health Network, Toronto, ON M5G 2C4, Canada; 3 Centre de Recherche du Centre Hospitalier de l’Université de Montréal, Montréal, QC H2X 0A9, Canada; gabrielle.page@umontreal.ca; 4 Department of Anesthesiology and Pain Medicine, Faculty of Medicine, Université de Montréal, Montréal, QC H3T 1J4, Canada; 5 Department of Anesthesia and Pain Medicine, Hospital for Sick Children, Toronto, ON M5G 1X8, Canada; lisa.isaac@sickkids.ca (L.I.); Fiona.campbell@sickkids.ca (F.C.); 6 Department of Anesthesiology and Pain Medicine, Temerty Faculty of Medicine, University of Toronto, Toronto, ON M5S 1A8, Canada; 7 Department of Anesthesia and Pain Medicine, Child Health Evaluative Sciences Research Institute, Hospital for Sick Children, Toronto, ON M5G 1X8, Canada; Jennifer.stinson@sickkids.ca; 8 Lawrence S Bloomberg Faculty of Nursing, University of Toronto, Toronto, ON M5T 1P8, Canada; 9 Department of Psychology, Hospital for Sick Children, Toronto, ON M5G 1X8, Canada

**Keywords:** pain, chronic pain, children, adolescents, parents, functional limitations, functional disability, anxiety

## Abstract

Pediatric chronic post-surgical pain is a surgical complication associated with various levels of functional limitation. Two commonly used measures of functional limitations in youth are the Functional Disability Inventory (FDI) and the PROMIS Pediatric Pain Interference Scale (PPIS), where the former is general, and the latter, pain specific. The aim of the present study was to prospectively compare pre-surgical youth and parent risk factors for youth functional limitations, assessed by the FDI and PPIS, 12 months after major pediatric surgery. Risk factors for the FDI and PPIS were compared in 79 dyads consisting of youth (58% female, *M* = 14.56 years; SD = 2.31) undergoing major surgery and one of their parents. The FDI and PPIS were highly correlated prior to surgery (*r* = 0.698, *p* < 0.001) and even more so 12 months after surgery (*r* = 0.807, *p* < 0.001). Parent pre-surgical anxiety sensitivity and youth pre-surgical functional disability significantly predicted 12-month FDI (*F*(6,56) = 4.443, *p* = 0.001, Adjusted R^2^ = 0.25), whereas parent pre-surgical anxiety sensitivity, trait anxiety, pain anxiety, as well as youth pain-related anxiety and worry significantly predicted 12-month PPIS (*F*(6,45) = 4.104, *p* = 0.002, Adjusted R^2^ = 0.27). Risk factors for 12-month general and pain-specific functional limitations differ by dyad member and type. Functional limitations in youth after surgery are predicted by youth and parent factors, however the risk factors differ between the FDI and the PPIS.

## 1. Introduction

Chronic post-surgical pain is a surgical complication reported by 11–54% of children and adolescents after major surgery [1,2,3,4]. Youth with moderate-to-severe chronic pain are often limited in their age-appropriate everyday activities, such as being social with friends, going to school, and doing physical activity [4,5,6,7].

The interpersonal fear-avoidance model of pain (IFAM; [8]) uses a family systems perspective that identifies child (e.g., cognitive, affective, behavioral) and parent (e.g., cognitive, affective, behavioral), parent-child dyad (e.g., parent-child interaction style), and family (e.g., familial environment) factors instrumental in the development and maintenance of functional limitations (e.g., disability, interference) associated with chronic pain. There is evidence in support of the IFAM model from cross-sectional studies showing bi-directional relationships among child chronic pain-related disability and child and parent, parent-youth dyad, and family variables [9,10]. However, this relationship has rarely been evaluated longitudinally. A recent systematic review and meta-analysis of youth with chronic pain [10] found only four prospective studies [11,12,13,14] to have evaluated parent and youth factors and their relationship to functional limitations over time using the Functional Disability Inventory (FDI), the Child Activities Limitations Interview (CALI), and the Child Health Assessment Questionnaire (CHAQ). To our knowledge, none has evaluated these relationships in the transition of acute to chronic postsurgical pain.

The Pediatric Initiative on Methods, Measurement, and Pain Assessment in Clinical Trials (PedIMMPACT) consensus guidelines recommend the use of measures to evaluate functional limitations in children with chronic pain [15]. Until recently the FDI [16] was the measure recommended for assessment of general physical functioning in children with chronic pain [15]. More recently the Patient-Reported Outcomes Measurement Information System (PROMIS) developed the short Pediatric Pain Interference Scale (PPIS) designed to evaluate pain-specific functional limitations [17]. These two scales measure similar aspects of functioning; however, they differ in that the FDI is a general measure of functional limitations that does not refer to pain but rather captures social, developmental, and other medical problems, whereas the PPIS is a pain-specific measure. However, they are often used interchangeably in outcome research. The FDI and the PPIS are well validated and widely used measures of functional limitations, but it is unclear what, if any, youth and parent factors differentially predict their outcomes in the transition from acute to chronic pain. This is important for two reasons. First, the FDI and PPIS are conceptually distinct measures, and as such are likely to be impacted by different variables. Second, if there are differences in risk factors, there may be implications for how best to intervene. The present study was designed to prospectively compare pre-surgical youth and parent risk factors for the PPIS and the FDI 12 months after major pediatric surgery. Due to the high level of parent involvement in youth surgical recovery, we hypothesize that parent risk factors will predict youth functional limitations 12 months after surgery. We hypothesize that parent general anxiety will predict general functional limitations (FDI), whereas parent pain-related anxiety will predict pain-related functional limitation (PPIS). Further, we hypothesize that youth general anxiety will be associated with general functional limitations (FDI) and that youth pain-related anxiety will be associated with pain-related functional limitations (PPIS). Secondarily, this study aimed to examine the association between chronic pain status and functional limitations (FDI, PPIS).

## 2. Materials and Methods

The present article is part of a larger study evaluating the incidence of chronic post-surgical pain in children and adolescents (youth) [4]. The methods and results reported herein pertain only to the objective of the present article, which was to evaluate the differential pre-operative risk factor profiles of pediatric pain-related functional limitations and general functional limitations 12 months after surgery. Therefore, data are presented only from Time 0 (T0) corresponding to the pre-operative assessment and Time 3 (T3) corresponding to the 12-month post-operative assessment.

### 2.1. Participants

Participants were eligible for the present study if they were 8–18 years of age, undergoing either orthopedic surgery or general surgery, and one of their parents were agreeable. Exclusion criteria for youth were as follows: (1) documented developmental or cognitive delay, (2) cancer diagnosis, (3) could not speak or read English, or (4) their parent/guardian did not speak or read English.

### 2.2. Questionnaires

#### 2.2.1. Outcome Measures

Pain-related interference, or pain-related functional limitation, was measured by the PROMIS—Pediatric Pain Interference Scale (PPIS). The PROMIS—Pediatric Pain Interference Scale [17] is an 8-item, self-report instrument that measures the extent to which, over the “past 7 days”, the youths’ pain has interfered with, or limited them from engaging in, important functional facets of their everyday life (e.g., emotions, physical activities, schoolwork, attention, sleep). Participants use a 5-point scale that ranges from “never” to “almost always” to rate each item. Total possible scores range from 0 to 32 with higher scores indicating more pain-related interference or greater pain-related functional limitations. Raw scores are transformed to t-scores. The PPIS consistently achieves a reliability of 0.85 [17,18]. T0 (α = 0.93) and T3 (α = 0.92) internal consistency for the present study were excellent.

General functional disability, or general functional limitation, was measured by the Functional Disability Inventory (FDI). The 15-item FDI [16] measures how much difficulty youth report in carrying out daily tasks and activities (e.g., “Being at school all day”, “Eating regular meals”, and “Walking to the bathroom”). Items are rated on a 5-point Likert Scale (0 = “no trouble” to 4 = “impossible”. FDI total possible scores range from 0 to 60 with higher scores indicating greater general functional limitations or increasing difficulty with the tasks and activities. The FDI has very good to excellent psychometric properties, including very strong internal consistency (α = 0.90) and good concurrent validity [16]. FDI T0 (α = 0.92) and T3 (α = 0.91) internal consistency were excellent.

#### 2.2.2. Youth Risk Factor Measures

Pain was measured using a Numerical Rating Scale (NRS). The 11-point NRS is 0–10 verbal scale that measures participants’ self-reported experience of pain intensity (NRS-I) or pain unpleasantness (NRS-U). The NRS-I ranges from 0 = “no pain at all” to 10 = “worst possible pain”. The NRS-U ranges from 0 = “not at all unpleasant/horrible/yucky”) to 10 = “most unpleasant/horrible/yucky”. The NRS has very good to excellent psychometric properties, including at least adequate reliability, construct validity, and sensitivity to change over time in youth (7–18 years) with acute postoperative pain after a variety of surgical procedures [19,20].

Pain thresholds were measured using Pressure Algometry. A Pressure Algometer (Baseline^®^ Dolorimeter, Model PR0379 and PR0376, Algeos Ltd., Liverpool, UK) was used to obtain pressure thresholds. The algometer is a hand-held device with a 1.5 cm rubber tip attached to a spring-loaded gauge that displays the force applied in kg/sq cm. The algometer was applied a rate of ~0.5 kg/s to a point on the skin over muscle. Pressure pain threshold (PPT) was defined as the applied force (pressure/unit area) corresponding to when the participant first reported pain after which they rated the intensity of the pain using an 0–10 NRS. Baseline PPTs were obtained before surgery (at the pre-admission visit), at the proposed incision site (or sites) and at control sites on the left and right forearm (anterior aspect midway between the elbow and wrist). Pressure algometry has been used and validated in children with orthopedic conditions [21].

Pain acceptance was measured using the following self-report instruments: the Chronic Pain Acceptance Questionnaire-Adolescents (CPAQ-A) and the Child Self-Efficacy Scale-Child Version (CSES-C). The CPAQ-A [22] is a 20-item scale that measures an adolescent’s acceptance of chronic pain. The internal consistency for the activity engagement subscale has been shown to be good (α = 0.86) and also adequate for pain willingness (α = 0.75) [22]. Internal consistency for the present study was very good at T0 (α = 0.88). The CSEC-C [23] is a 7-item measure of a child’s belief that they can engage in specific activities, such as going to school, taking care of him/herself, and participating in activities with family or friends, without assistance. The CSES-C has good internal consistency (α = 0.80 to 0.83) [23]. Internal consistency for the present study was very good at T0 (α = 0.88).

Pain-related anxiety and worry was measured using the following self-report instruments: the Pain Catastrophizing Scale—Children (PCS-C [24]), the Child Pain Anxiety Symptoms Scale (CPASS), and the Tampa Scale for Kinesiophobia (TSK-13). The PCS-C is a child and adolescent version of the 13-item PCS [25]. The PCS-C measures catastrophic thinking about pain, including unrealistic thoughts that the worst possible outcome will arise, negative thoughts about the self and future, and “an exaggerated negative ‘mental set’ brought to bear during actual or anticipated pain experience” (p. 53, [26]). PCS-C internal consistency (α = 0.90) is excellent, and it shows moderate correlations with pain intensity (r = 0.49) and pain disability (r = 0.50) [24]. T0 PCS-P internal consistency (α = 0.94) in the present study was excellent. The 20-item CPASS [27] measures the thoughts, feelings, behaviors, and physical sensations that arise during the experience and expectation of pain. CPASS has excellent internal consistency (α = 0.89 to 0.90) and strong construct validity [27,28,29]. T0 CPASS internal consistency (α = 0.92) in the present study was excellent. The 13-item TSK-13 measures fear of movement-evoked pain and injury or re-injury. It has acceptable psychometric properties, including good discriminative and predictive validity and adequate internal consistency [30]. T0 TSK-13 internal consistency (α = 0.81) in the present study was good.

General anxiety and worry were measured using the following self-report instruments: Multidimensional Anxiety Scale for Children (MASC-10), the Children’s Revised Impact of Event Scale (CRIES; [31,32]), and the Childhood Anxiety Sensitivity Index (CASI). The 10-item MASC-10 [33] is a short version of the 39-item MASC-39. The MASC-10 measures physiological reactions, harm avoidance, and symptoms of social anxiety and separation anxiety. The MASC-10 has excellent internal consistency (α = 0.89), strong test-retest reliability (r = 0.86), and good convergent and discriminant validity [33,34]. T0 MASC-10 internal consistency (α = 0.91) for the current study was excellent at. The 13-item CRIES measures symptoms of posttraumatic stress disorder (PTSD) reported over the past six months. The CRIES has good reliability (α = 0.80) [32] and strong validity when screening for PTSD [35]. T0 CRIES internal consistency (α = 0.91) for the present study was excellent. The 18-item CASI [36] measures how extensively the symptoms of anxiety (e.g., rapid heart rate, rapid breathing, racing thoughts) evoke fear and beliefs about ensuing harmful somatic, psychological, and social consequences. The CASI has very good internal consistency (α = 0.87), adequate test-retest reliability (r = 0.76) and satisfactory construct validity [36]. T0 CASI internal consistency (α = 0.86) for the present study was very good.

Depressive symptoms were measured using the Center for Epidemiological Studies-Depression Scale for Children (CES-DC). The CES-DC assesses depressive symptoms in children and adolescents, including depressed or low mood, a sense of worthlessness, helplessness, psychomotor retardation (slowed speaking, thinking, movement), as well as sleep and eating problems. The CES-DC has excellent internal consistency (α = 0.89) and good convergent validity [37]. Internal consistency for the present study was excellent at T0 (α = 0.92).

#### 2.2.3. Parent Risk Factor Measures

Parental pain-related anxiety and worry was measured using the following self-report instruments: the Pain Anxiety Symptoms Scale—Short Form (PASS-20), the Pain Catastrophizing Scale (PCS), and the Pain Catastrophizing Scale—Parents (PCS-P). The 20-item PASS-20 [38] measures the thoughts, feelings, behaviors, and physical sensations that arise during the experience and expectation of pain. Internal consistency (α = 0.81) and construct validity for the PASS-20 are good. PASS-20 internal consistency (α = 0.95) for the present study was excellent. The 13-item PCS [25] measures catastrophic thinking about pain, including unrealistic thoughts that the worst possible outcome will arise, negative thoughts about the self and future, and “an exaggerated negative ‘mental set’ brought to bear during actual or anticipated pain experience” (p. 53, [26]). The PCS measures an individual’s catastrophic thinking about their own pain. The PCS has very good internal consistency and validity [39]. The PCS-P [40] is the parent version of the PCS that measures a parent’s catastrophic thinking about their child’s pain. Construct validity and internal consistency (α = 0.81 to 0.93) [40] of the PCS-P are very good. T0 internal consistency for the PCS (α = 0.94) and PCS-P (0.92) in the present study were excellent.

Parental anxiety and worry were measured with the following self-report instruments: the Anxiety Sensitivity Index (ASI) and the State-Trait Anxiety Inventory-Trait (STAI-T). The ASI [41] measures how extensively the symptoms of anxiety evoke fear and beliefs about ensuing harm. The ASI consists of three subscales: fear of publicly observable anxiety reactions (4 items, e.g., “It embarrasses me when my stomach growls”); fear of cognitive dysfunction (4 items, e.g., “It scares me when I am unable to keep my mind on a task”); and fear of somatic sensations (8 items; e.g., “It scares me when my heart beats rapidly”). The ASI total score has very good internal consistency (α = 0.83) and good convergent and discriminant validity [42]. Internal consistency for the present study was very good at T0 (α = 0.90). The STAI-T [43] is a 20-item scale that measures a wide range of enduring, not state or momentary, symptoms of anxiety. It has been shown to have very good to excellent internal consistency (α = 0.86 to 0.95) and satisfactory test-retest reliability (r = 0.69 to 0.89) [43]. STAI-T construct and concurrent validity are good [44]. Internal consistency for the present study was excellent at T0 (α = 0.92).

Parental symptoms of depression were measured using the Center for Epidemiological Studies-Depression Scale (CES-D). The CES-D [45] is a 20-item scale for adults that measures depressive symptoms, such as depressed or low mood, a sense of worthlessness, helplessness, psychomotor retardation (slowed speaking, thinking, movement), as well as sleep and eating problems. CES-D has shown to have high internal consistency (α = 0.85 to 0.90) and strong construct validity [45]. Internal consistency for the present study was good at T0 (α = 0.80).

Parental psychological flexibility was measured with the Parent Psychological Flexibility Questionnaire (PPFQ). The PPFQ [46] measures the parents’ capacity to manage their distress about their child’s pain. The PPFQ has excellent internal consistency (α = 0.91) [46]. Internal consistency for the PPFQ at T0 (α = 0.89) in the present study was good.

### 2.3. Procedure

The Research Ethics Board at The Hospital for Sick Children (SickKids) (REB file # 1000019644) and the Human Participants Review Committee at York University (Certificate # 2010-276) reviewed and approved the protocol before recruitment began. A research assistant approached eligible and interested youth and parent dyads after they had been pre-screened by an operating room nurse within in the youths’ circle of care and had agreed to hear more about the study. Youth and their parents were recruited into the study by the research assistant either at the pre-operative assessment clinic or, if they did not attend the pre-operative clinic, by telephone. Parents and youth provided written informed consent and assent to participate, respectively.

This prospective, longitudinal study comprised two assessments over a one-year period: pre-operative, and 12 months after surgery. Participants were included in analysis for the present study if they completed both the PPIS and the FDI. We focused on the 12-month post-surgical outcome to evaluate chronic functional limitations.

#### 2.3.1. Pre-Operative Assessment (T0)

The research assistant administered the baseline assessment youth and parent questionnaires and obtained pressure pain thresholds from the participants using pressure algometry. The sequence in which questionnaires were administration was randomized within subjects to reduce the effects of order and fatigue effects. Youth pre-operative medication use was ascertained from the parents and verified by accessing the youths’ hospital chart.

#### 2.3.2. 12 (T3) Month Post-Surgical Follow-Up

The research assistant conducted telephone follow-ups with participants 12 months after surgery. Participants completed a package of questionnaires and inventories that measured pain, psychological and emotional functioning, memory for pain, pain medications, chronic postsurgical pain (i.e., pain incidence, intensity, and quality), as well as general and pain-related function limitations. At the 12-month follow-up with the parents, the research assistant read parents, over the telephone, two questionnaires that assessed psychological flexibility and catastrophic thinking about their youth’s pain.

### 2.4. Statistical Analysis

Data were analyzed using the Statistical Package for the Social Sciences (SPSS) version 24.0 and R version 3.4.1. Descriptive, correlational, and regression analyses were conducted using two-tailed hypothesis testing (*p* < 0.05). Descriptive statistics, including frequency tables, means, and standard deviations were used to describe sample characteristics. Bivariate relationships between variables were analyzed using Pearson’s *r*. Paired samples t-tests were conducted to compare T0 PPIS to T3 PPIS, and T0 FDI to T3 FDI. We tested for collinearity among variables.

The association between chronic pain status (i.e., T3 presence of chronic post-surgical pain) and T3 FDI was examined through a logistic regression. This approach was repeated for T3 PPIS.

A set of univariate General Linear Models (GLMs) were used to examine the association between risk factors (youth pain intensity, youth pain unpleasantness, youth pain pressure threshold, youth pain-related anxiety and worry, youth non-pain related anxiety and worry, youth depression, youth pain acceptance, and parental pain-related anxiety and worry, parental non-pain related anxiety and worry, parent depression, and parent psychological flexibility) and youth outcome variables at 12 months (PPIS, FDI).

Two sets of identical hierarchical regression analyses were conducted, one predicting 12-month PPIS scores and the other predicting 12-month FDI scores. Each set evaluated three models using the same significant (*p* < 0.05) child and parent risk factors identified from the univariate linear regression analyses. Risk factors were chosen based on statistical significance and theory (i.e., IFAM). Model 1 included an autoregressive term to control for baseline FDI or PPIS, respectively. Since the aim of the study is to determine differential risk factors for functional limitation, not to examine whether one measure of functional limitation predicts another measure of functional limitation, we did not include both measures of functional limitation in each model. Model 2 included Model 1 predictors plus baseline youth factors. Model 3 included Model 2 variables plus baseline parent factors. The overall performance of the multiple regression models was evaluated based on adjusted R^2^ and Δ R^2^. The order of the models was chosen to evaluate the variance accounted for by youth baseline psychological factors over and above their baseline functioning, as well as the variance accounted for by parent psychological factors over and above the youth variance predicting youth functional limitations 12-months after surgery.

## 3. Results

### 3.1. Recruitment and Demographic Information

Recruitment occurred between February 2011 and August 2015. Details about the recruitment procedures have been described in earlier publication [4,47]. Of the 349 children and parents approached, 270 gave their assent and informed written consent to participate, respectively. Three children retracted consent before the start of the study procedures and did not participate in any way, one patient no longer met the study criteria after the surgical team changed the surgical procedure, and 27 children could not be found (i.e., the research assistant was unable to locate or reach them) for their T0 assessment. One participant was withdrawn from the study after informed consent had been obtained because they received a cancer diagnosis. Two hundred and sixty-four (264) participants successfully finished at least one part of the hospital-based assessment (T1) (e.g., completed questionnaires, wore the Actical physical movement monitor, provided daily measures of pain). Twenty-seven (27) participants were admitted to the intensive care unit (ICU) directly from the operating room (i.e., they bypassed the PACU) and consequently daily pain measures were not obtained, nor was the Actical device secured on the participants’ wrist until they had been moved out of the ICU to a surgical floor. More than 80% (81.1% and 85.3%) of participants were successfully followed up at the 6- and 12-month assessments, respectively.

The PPIS was included in the study questionnaire package in 2013. A total of 79 patients completed the PPIS before surgery and after surgery. Therefore, the analysis for this study was conducted on this subsample of patients who completed the PPIS. The sample comprised 79 children [46 female (58.22%), *M* age = 14.56 years (SD = 2.31), range 9–18 years] and their parents/guardians [61 female (84.72%), M age = 45.82 years (SD = 6.76), range 30–70 years, 7 parents did not respond to the demographic questionnaire items]. Half of the children had moderate-to-severe pain prior to surgery (*n* = 40, 50.63%). Fewer than 33%of parents had ongoing pain issues before their child’s surgery (*n* = 21, 28%). Just under 50% of children had surgery for scoliosis (*n* = 39, 49.37%) and 41.77% (*n* = 33) had an osteotomy. Five children (6.33%) had a Ravitch sternotomy procedure for correction of pectus excavatum and two (2.53%) had another type of surgery. The mean duration of surgery was 4.76 h (SD = 1.95 h, range = 1.03–9.25 h) and children remained hospitalized for an average of 4.77 days (SD = 2.08, range 1–11 days). Twenty-four percent (*n* = 19) of youth had regional anesthesia for their surgery. Detailed demographic and Actical results for the sample are published in Rosenbloom et al. [4].

### 3.2. Relationship between PPIS and FDI

The FDI and the PPIS were significantly correlated before surgery (*r* = 0.698, *p* < 0.001) and 12 months after surgery (*r* = 0.807, *p* < 0.001). The mean score of the PPIS before surgery was significantly higher (M = 16.18, SD = 9.95) than 12 months after surgery (M = 11.65, SD = 8.30), *t*(57) = 2.564, *p* = 0.013. Maximum score on the PPIS is 32. In contrast, the mean score of the FDI before surgery (M = 13.80, SD = 12.24) was not significantly different than the mean FDI score 12 months after surgery (M = 12.81, SD = 11.19), *t*(70) = 0.665, *p* = 0.508. Maximum score on the FDI is 60.

The 12-month FDI was significantly associated with 12-month pain status (OR = 1.051, 95% CI 1.003, 1.102), in that every unit increase on the FDI was associated with increased odds that the youth had moderate to severe pain. The 12-month PPIS was significantly associated with 12-month pain status (OR = 1.066, 95% CI 1.018, 1.115), in that every unit increase on the PPIS was associated with a greater odds of the youth having moderate to severe pain.

### 3.3. Risk Factors for 12-Month PPIS and FDI

There was a high degree of collinearity (VIF > 4) between the youth MASC-10, CRIES, CASI, PCS, CPASS, and CSESC. To address this, we performed an Exploratory Factor Analysis on the child variables in R Version 3.4.1 [48] using the packages “car” [49], “GPArotation” [50], and “psych” [51]. Using ordinary least squares (OLS) to find the minimum residual (minres) solution, the number of factors for the child variables were evaluated. The number of factors was determined by an examination of a parallel analysis. The analysis revealed a two-factor model fit the data best, with all factor loadings >0.4 and cross-loadings <0.4. The factor loadings showed that Factor 1 (pain-related psychological factor) consisted of the PCS, CPASS, and CSESC and that Factor 2 (general psychological factor) consisted of the CASI, MASC-10, and CRIES. These factors were used in the linear regression analyses.

To determine significant risk factors for 12-month PPIS and FDI, univariate regression analyses were undertaken (Table 1). Significant baseline factors associated with 12-month PPIS included: youth pain at rest, youth TSK-13, youth pain-related psychological factor, youth general psychological factor, and youth FDI. Significant factors associated with 12-month FDI included: youth pain-related psychological factor, youth TSK-13, parent ASI, parent STAI-T, parent PASS, youth FDI, and youth PPIS.

We separately examined three identical models for each outcome variable. Model 1 predictors included baseline youth FDI or youth PPIS aligned with 12-month FDI or 12-month PPIS as dependent variables, respectively. Model 2 predictors included Model 1 variables as well as youth pain-related anxiety and worry factor and youth general psychological factor. Model 3 predictors included Model 2 variables as well as parent ASI, parent PASS, and parent STAI-T. The results from the hierarchical regression models for the FDI and PPIS are shown in Table 2 and Table 3, respectively.

Prediction of 12-month youth FDI: Pre-surgical youth FDI significantly predicted 12 month FDI (Model 1), *F*(1, 61) = 14.937, *p* < 0.001, R^2^ = 0.184. The addition of the two youth factors to Model 2, *F*(3, 59) = 5.194, *p* = 0.003, R^2^ = 0.169, did not add a significant proportion of variance to the prediction model, ΔR^2^ = 0.012, *p* < 0.636. However, the addition of pre-surgical parent variables, in Model 3, explained an additional 11.4% (ΔR^2^ = 0.114, *p* = 0.033) of the variance in 12-month FDI scores, *F*(6, 56) = 4.443, *p* = 0.001, R^2^ = 0.250. Thus, for Model 3, every unit (standard deviation) increase in pre-surgical youth FDI was associated with a 0.372 unit increase in their child’s functional disability 12 months after surgery. And every unit (standard deviation) increase in pre-surgical parent ASI was associated with a 0.373 unit increase in their child’s functional disability 12 months after surgery. None of the other risk factors significantly predicted 12-month FDI.

Prediction of 12-month youth PPIS: Pre-surgical youth PPIS did not significantly predict 12-month PPIS (Model 1), F(1, 50) = 0.397, *p* = 0.531, R^2^ = −0.012. Model 2, including the two youth factors, significantly predicted 12-month PPIS F(3, 48) = 3.902, *p* = 0.014, R^2^ = 0.146. Youth pre-surgical pain-related anxiety and worry factor was significantly associated with 12-month youth PPIS, such that every unit (standard deviation) increase in the factor there was an associated 0.448 increase in 12-month PPIS. Finally, the addition of pre-surgical parent factors to Model 3 explained an additional 15.8% of the variance, *p* = 0.019, in 12-month PPIS scores, F(6, 45) = 4.104, *p* = 0.002, R^2^ = 0.267. Thus, for Model 3, every unit (standard deviation) increase in pre-surgical parent ASI was associated with a 0.401 unit increase in their child’s pain-related interference 12 months after surgery. Similarly, every unit (standard deviation) increase in pre-surgical parent STAIT was associated with a 0.403 unit increase in their child’s pain-related interference 12 months after surgery. And for every unit (standard deviation) increase in parent pre-surgical PASS-20 there was a 0.617 decrease in their child’s pain-related disability at 12-months post-surgery.

## 4. Discussion

The present study evaluated differential baseline risk factors in the development of general versus pain-specific functional limitations 12 months after major pediatric surgery. The results show that although the FDI and PPIS are highly correlated, and the strength of their inter-relationship increases over time, the risk factors for general versus pain-specific functional limitations differ. Specifically, 12-month FDI is predicted by pre-surgical child FDI and parent anxiety sensitivity whereas PPIS is predicted by a combination of presurgical youth (pain-related anxiety and worry factor) and parent factors (anxiety sensitivity, state-trait anxiety, pain anxiety). In line with our hypothesis, hierarchical regression analysis showed that the addition of parent factors to Model 3 was significant in predicting both the PPIS and FDI; qualitatively more so for the PPIS than the FDI in that whereas both functional limitation measures were predicted by parent ASI, the PPIS was also predicted by parent trait anxiety and pain-related anxiety.

Increasingly, chronic pain and associated disability among youth has been identified as family issues [10,52] associated with intergenerational transmission [52]. The results of the present study support this suggestion showing that general and pain-specific functional limitations associated with 12-month pediatric CPSP are predicted by parental factors measured preoperatively, one year earlier. Until recently, many studies have focused on parent chronic pain as a risk factor for youth pain interference [52,53]. However, the present results did not confirm the links between the presence of parent chronic pain and youth post-operative pain-related functional limitations or general functional limitations. This is perhaps due, in part, to the nature of the chronic pain (i.e., general chronic pain (e.g., arthritis, headaches) pain versus CPSP). Instead, the present results indicate that parental pre-surgical anxiety (both pain- and non-pain related) are important in the development of youth functional limitations one year after surgery.

The identification of parental anxiety sensitivity as a significant predictor of both youth 12-month pain-related functional limitations (PPIS) and general functional limitations (FDI) is a novel finding. The results suggest that parents’ with higher levels of anxiety sensitivity may shape the development of their youth’s functional limitations over the first year after surgery. The adult literature proposes that anxiety sensitivity amplifies fear, anxiety, and subsequently increases the likelihood to engage in avoidance or escape behaviors (e.g., limit movement) in response to pain [54,55]. We propose that parents with elevated levels of anxiety sensitivity may be on high alert for signs of pain or anxious distress in their offspring. In high anxiety sensitive parents, the ensuing anxiety triggers alarm and so they search for ways to avoid or escape from their own anxiety by removing the cause of their anxiety and encourage avoidance behaviors in their offspring (e.g., encouraging their child to functionally limit their behaviors and activities).

Pre-operative child functional disability was the only child factor that predicted 12-month post-surgical general functional limitations (FDI). In contrast, pre-surgical pain-related functional limitations did not predict 12-month pain-related functional limitation (PPIS). It is not surprising that the best predictor of functional disability 12-months after surgery was pre-operative functional disability; what is surprising is the lack of a relationship between pre-surgical PPIS scores and 12-month PPIS scores. The discrepancy in predictors for the two outcome measures may be related to the general versus pain-specific nature of the FDI and PPIS, respectively. It is possible that since most youth had not previously experienced pain as intense as that arising from surgery the youths’ baseline level of pain-related interference changed as a function of exposure to the intense acute postsurgical pain experience, so that by the 12-month post-surgical assessment, their pre-surgical scores no longer accurately reflected their pain experiences, thereby explaining the lack of a relationship between pre- and post-surgical PPIS scores.

Catastrophic thinking about pain is an important factor in the adult and pediatric chronic pain literature. Both child and parent pain catastrophizing have been shown to have correlations with child chronic pain functional outcomes [24,40,56,57,58]. Among children and adolescents with chronic pain, the extant literature supports the idea that parent catastrophic thinking about (their own) pain predicts child functional limitation through their child’s catastrophic thinking about pain [59] or adolescent psychosocial responses to pain [60]. Due to the multicollinearity between youth pre-operative measures of pain catastrophizing, pain anxiety and self-efficacy, the individual effects of each measure were not examined on 12-month functional limitations. Given that the pain-related anxiety and worry construct did significantly contribute to 12-month PPIS, it is possible that youth pain catastrophizing played a role in this outcome, but not 12-month FDI. We did not find that pre-surgical parent pain catastrophizing predicted 12-month outcomes and thus our results suggest that parent pre-operative pain catastrophizing is not a significant risk factor involved in functional limitations associated with the transition from acute to chronic pain in youth undergoing surgery. This, again, may be related to the timing of measurement of pain catastrophizing relative to surgery, a point we raised [61] regarding a similarly designed study by Rabbitts et al. who also did not find that pre-operative child catastrophic thinking about pain predicted pain outcomes after surgery [62]. As with pre-surgical PPIS scores which did not predict 12-month PPIS scores, we argue that the child’s baseline level of catastrophic thinking about pain may have changed after exposure to the intense acute postsurgical pain, so that by postoperative assessment, the youths’ preoperative pain catastrophizing scores no longer accurately reflected their pain experiences. The issue of changing baselines is critical to accurate prediction and could be better studied and understood using qualitative designs to get at the psychosocial mechanisms involved in this process.

Resilience factors for both parent and youth, such as pain acceptance and self-efficacy, also were not significant predictors of either 12-month FDI or PPIS in our study. There are no known studies examining these factors in pediatric surgical samples. Compared to the pediatric chronic pain literature, our results are contrary to Feinstein et al., who studied a sample of pediatric patients with chronic pain (e.g., musculosketal pain, abdominal pain, headache, complex regional pain syndrome) and found that parent pain acceptance was associated with pain interference through child pain acceptance [63]. Several factors that make it difficult to compare the results of the two studies. For example, Feinstein et al. conducted a cross-sectional study of children at the initial visit to a chronic pain clinic. Children reported chronic pain of approximately 30 months’ duration and various etiologies. In contrast, the present sample of youth with chronic postsurgical pain was followed prospectively for up to one year. Thus, it possible that differences in the etiology and duration of the pain, as well as in the design and clinical setting may in part be responsible for the difference between the two studies in resilience factor outcomes.

In addition to youth pre-surgical functioning, the results from this study show that it is critically important to consider parental risk factors critical in the development of youth post-surgical general and pain-related function limitations one year after surgery. Parental anxiety and anxiety sensitivity are potentially modifiable variables through, for example, Acceptance and Commitment Therapy (ACT) [64,65] or Cognitive-Behavioural Therapy (CBT) [66]. Through ACT, it is possible that parents can learn to think more flexibly about pain and accept what they struggle to control regarding their youth’s pain, and as a result their youth’s functional limitation outcome could shift positively. It has been shown that caregivers who endorse greater pain acceptance engage in less catastrophic thinking [67], which indirectly may increase functioning [68]. Further it is possible through directly treating anxiety sensitivity using a 1-session CBT approach [66], parents will learn to cope with their own anxiety sensitivity, which would in turn help them coach their children on how to manage pain.

Study strengths include the prospective methodology used to examine the risk factors for the transition from acute to chronic post-surgical pain and related functional limitations versus pain-specific functional limitations. This study showed that there are different risk factors associated with general versus pain-specific functional limitations and therefore researchers should thoughtfully consider whether they are mainly studying general or pain-specific functional limitations.

There are several limitations to note. First, the sample size was relatively small, limiting potential generalization. Hence, replication in a larger sample is warranted. Likewise, more sophisticated statistical analyses, such as structural equation modeling, could not be performed to examine indirect relationships between parent anxiety and anxiety sensitivity and child pain interference outcomes. Second, this is a secondary analysis, which raises two potential issues: a priori sample size calculations were not conducted for this outcome and therefore there is the possibility of a high risk of bias. Third, it is possible that potentially confounding variables for these secondary analyses were not measured. The analyses indicated that 25% of the variance for the FDI was explained by the model factors leaving 75% unexplained. Similarly, the analyses indicated that 26.7% of the variance for the PPIS was explained by the model factors, leaving 73.3% unexplained. Although we do not know what factors make up the unexplained variance in each set of analyses, future studies should consider evaluating parent factors such as solicitousness and pain resilience, and child factors such as psychological flexibility.

## 5. Conclusions

In conclusion, although the FDI and the PPIS are highly correlated and used widely in the literature, they represent different constructs and are predicted by slightly different risk factors in the transition from acute to chronic pain and related disability. This underscores the need for researchers and clinicians to carefully consider the construct of interest when choosing a measure. In terms of youth factors, pre-operative youth general functional limitation uniquely predicts post-surgical general functional limitation. Additionally, pain-related anxiety and worry and general psychological anxiety and stress predict 12-month pain-related functional limitation. In terms of parental involvement in the development of disability, we provide support for the inclusion of parental risk factors in studying the transition from acute to chronic pain and identify parental pre-operative anxiety and anxiety sensitivity as significant risk factors. These results suggest that parent anxiety plays a role in intergenerational processes in the development of pain-related interference and general functional disability after surgery and is therefore an area for assessment and intervention in future research.

## Figures and Tables

**Table 1:** Standardized regression coefficients (β) from univariate regression analyses predicting 12-month functional disability (FDI) and pain-related interference (PPIS) from pre-surgical parent and youth risk factors.

	Variable	12-Month Youth FDI β	12-Month Youth PPIS β
YOUTH	Age	−0.050	0.125
	Sex	0.182	0.085
	Pressure algometer	−0.160	−0.061
	NRS pain (rest)	0.175	0.254 *
	NRS pain unpleasantness	0.101	0.018
	TSK-13	0.237 *	0.323 **
	Pain-related anxiety and worry	0.279 *	0.301 *
	General anxiety and worry	0.173	0.320 **
	CES-DC	0.122	0.167
	CPAQ	−0.139	−0.090
	FDI	0.424 ***	0.331 **
	PPIS	0.330 **	0.131
PARENT	Chronic Pain	0.064	0.189
	PCS- about child pain	0.175	0.109
	PCS- about own pain	0.205	0.021
	CES-D	0.227	0.153
	ASI	0.313 **	0.146
	PASS	0.283 *	0.077
	STAI-T	0.237 *	0.199
	PPFQ	0.092	0.093

NRS, Numeric Rating Scale; TSK-13, 13-item Tampa Scale for Kinesiophobia; CES-DC; Center for Epidemiological Studies—Depression Scale for Children; CPAQ, Chronic Pain Acceptance Questionnaire—Adolescents; PPIS, PROMIS—Pediatric Pain Interference Scale; FDI, Functional Disability Index; PCS, Pain Catastrophizing Scale; CES-D, Center for Epidemiological Studies—Depression Scale; ASI, Anxiety Sensitivity Index; PASS-20, Pain Anxiety Symptoms Scale; STAI-T, State-Trait Anxiety Inventory-Trait; PPFQ, Parent Psychological Flexibility Questionnaire. Note: * *p* < 0.05; ** *p* < 0.01; *** *p* < 0.001.

**Table 2:** Standardized regression coefficients (β) from multivariable regression analyses predicting 12-month youth FDI from youth and parent pre-surgical risk factors.

	Adjusted R^2^	ΔR2	Pre-Surgical Variable	12-Month Youth FDI β
Model 1	0.18			
			Youth FDI	0.444 ***
Model 2	0.17	0.01		
			Youth FDI	0.382 **
			Youth pain-related anxiety and worry factor	0.117
			Youth General anxiety and worry factor	0.042
Model 3	0.25	0.11 *		
			Youth FDI	0.372 **
			Youth pain-related anxiety and worry factor	0.001
			Youth General anxiety and worry factor	−0.059
			Parent ASI	0.373 *
			Parent STAIT	0.159
			Parent PASS	−0.109

FDI, Functional Disability Index; ASI, Anxiety Sensitivity Index; PASS-20, Pain Anxiety Symptoms Scale; STAI-T, State-Trait Anxiety Inventory-Trait. Note: * *p* < 0.05; ** *p* < 0.01; *** *p* < 0.001.

**Table 3:** Standardized regression coefficients (β) from multivariable regression analyses predicting 12-month youth PPIS from youth and parent pre-surgical risk factors.

	Adjusted R^2^	ΔR^2^	Pre-Surgical Variable	12-Month Youth PPIS β
Model 1	−0.01			
			Youth PPIS	0.089
Model 2	0.15	0.19 **		
			Youth PPIS	−0.181
			Youth pain-related anxiety and worry factor	0.325 *
			Youth general anxiety and worry factor	0.337 **
Model 3	0.27	0.16 *		
			Youth PPIS	−0.244
			Youth pain-related anxiety and worry factor	0.448 *
			Youth General anxiety and worry factor	0.221
			Parent ASI	0.401 *
			Parent STAIT	0.403 *
			Parent PASS	−0.617 **

PROMIS—Pediatric Pain Interference Scale; ASI, Anxiety Sensitivity Index; PASS-20, Pain Anxiety Symptoms Scale; STAI-T, State-Trait Anxiety Inventory-Trait. Note: * *p* < 0.05; ** *p* < 0.01.

## Data Availability

Data are not available to share due to issues related to participant consent and REB restrictions.
